# Intermittent Hypoxic Training as an Effective Tool for Increasing the Adaptive Potential, Endurance and Working Capacity of the Brain

**DOI:** 10.3389/fnins.2022.941740

**Published:** 2022-06-21

**Authors:** Elena A. Rybnikova, Natalia N. Nalivaeva, Mikhail Y. Zenko, Ksenia A. Baranova

**Affiliations:** ^1^Pavlov Institute Physiology of Russian Academy of Sciences, St. Petersburg, Russia; ^2^I. M. Sechenov Institute of Evolutionary Physiology and Biochemistry of Russian Academy of Sciences, St. Petersburg, Russia

**Keywords:** hypoxia, adaptation, hypoxotherapy, intermittent hypoxic training, neuroprotection, periodic hypoxic training

## Abstract

This review is devoted to the phenomenon of intermittent hypoxic training and is aimed at drawing the attention of researchers to the necessity of studying the mechanisms mediating the positive, particularly neuroprotective, effects of hypoxic training at the molecular level. The review briefly describes the historical aspects of studying the beneficial effects of mild hypoxia, as well as the use of hypoxic training in medicine and sports. The physiological mechanisms of hypoxic adaptation, models of hypoxic training and their effectiveness are summarized, giving examples of their beneficial effects in various organs including the brain. The review emphasizes a high, far from being realized at present, potential of hypoxic training in preventive and clinical medicine especially in the area of neurodegeneration and age-related cognitive decline.

## Introduction

Adaptation to hypoxia is an extremely widespread event among living organisms, apparently being one of the most ancient evolutionary forms of adaptation (Bickler and Buck, 2007). The concept of hypoxia as an insufficient supply of oxygen to tissues and cells was initially associated only with pathological conditions (Meerson, 1984). However, these ideas underwent a significant transformation when it was shown that the state of hypoxia can also occur within “natural activity of the body” and, gradually, a conceptual transition was formed suggesting that hypoxia has both physiological significance for adaptive changes in response to the action of external hypoxic factors, and clinical significance for various pathophysiological conditions, especially cardio- and cerebrovascular diseases (for review see Hochachka, 1998). At the same time, the adaptogenic potential of hypoxia was successfully used in therapeutic strategies for prevention, rehabilitation, and treatment (Meerson et al., 1996; Rybnikova et al., 2008; Gonzalez-Rothi et al., 2015; Zenko and Rybnikova, 2019).

The adaptive reactions to physiological and exogenous hypoxia are of the same nature and have much in common with the compensatory and adaptive mechanisms in diseases accompanied by tissue hypoxia. Specific systemic mechanisms of adaptation to hypoxia include changes in lung ventilation, changes in the functioning of the cardiovascular system that enhance the delivery of oxygen to the tissues in need, and changes at the tissue level that allow more efficient use of oxygen for metabolic processes (Meerson et al., 1989; Serebrovskaya et al., 1999). The specific reactions to hypoxia are accompanied by an increase in the blood levels of glucocorticoids and this adaptive response ensures enhanced resistance not only to hypoxia but to many other environmental factors (Meerson et al., 1996; Zenko and Rybnikova, 2019).

The regulation of adaptive hypoxic response at the cellular level is largely due to the activation of hypoxia-sensitive transcription factors, in particular hypoxia-inducible factor 1 (HIF-1) which exists as a heterodimer of HIF-1α and HIF-1β subunits (Semenza, 2000). The oxygen level dose-dependently regulates the level of HIF-1α, which gradually increases following the decline of oxygen content in the air from 20 to 5% being particularly pronounced below 5% O_2_ (Jiang et al., 1996). More than 100 direct target genes have been identified for HIF-1, which cover erythropoiesis, angiogenesis, glucose transporters, as well as epigenetic machinery in the cells (for review see Corrado and Fontana, 2020; Kindrick and Mole, 2020). The dynamics of the HIF-1α level is characterized by rapid changes, both its increase and decline: for example, signs of HIF-1α decay after lung tissue reoxygenation appear in less than 1 min (Yu et al., 1998). Such rapid dynamics allows timely adaptive response to episodes of short-term hypoxia, which is especially important in the case of periodic hypoxia, and HIF-1, apparently, is a critical factor in initiating and reversing adaptive reactions to periodic hypoxia.

## History of the Hypoxic Training Implementation

One of the first written records of hypoxic therapy belongs to Hippocrates (430–370 BC), who recommended that patients move to live at a moderate altitude whereas the traveler Marco Polo (1,254–1,324) noted that inhabitants of Asia, when they are ill, go to the mountains to recover. The effects of moving to the mountain can be attributed to a special case of adaptation, acclimatization, which is an individual reaction of the body during a long stay in certain natural and climatic conditions. The duration of the acclimatization period depends on the altitude of the mountainous terrain, the temperature regime and the individual characteristics of the human body and can take from 7 to 25 days. Shorter periods of hypoxia (minutes-hours) alternating with periods of normoxia are called periodic or intermittent hypoxia. Experiencing this type of hypoxia has become more common in humans with the industrial development, progress of aviation and space industry, the development of high-altitude territories for economic activity, all of which played an important role in the need for a thorough study of the effects of periodic hypoxia on humans.

In the 1870s the French zoologist and physiologist Paul Bert (1833–1886) conducted around 700 experiments related to the physiological effects of altered atmospheric pressure, studying the condition of balloonists flying at different altitudes (Bert, 1878). Based on the results of the 1911 mountain expedition by John Scott Haldane (1860–1936) and studies in a low-pressure chamber down to 300 mm Hg (Haldane et al., 1919), suits for pilots and recommendations for improving adaptation to hypoxia were developed. In 1919 a chemical engineer Harold Pierce developed a pressure chamber equipped with a refrigeration unit, which allowed researchers to study the human reaction to a combination of cold and low atmospheric pressure (Jenkins, 2012).

In the 1980s Russian scientists proposed a concept of imitating mountain and pressure chamber hypobaric protective exposures by inhaling a gas mixture with reduced oxygen content at normal atmospheric pressure (Chizhov and Strelkov, 1992). Based on this concept, the method of intermittent hypoxic training (IHT) was developed (Serebrovskaya, 2002). Currently a large amount of clinical and experimental materials has been accumulated in the scientific literature describing the high effectiveness of IHT in medicine, including military, sport and wellbeing (Burtscher et al., 2010). IHT is also effectively used for preadaptation of troopers to operations in highlands or training of military or civil pilots (Muza, 2007; Neuhaus and Hinkelbein, 2014; Leinonen et al., 2021). Many hypoxic training regimens with periodic hypoxia were developed, with proven effectiveness in the treatment of cardiovascular, metabolic, neurological disorders, allergies and bronchial asthma, diabetes, and many other common diseases (Powell and Garcia, 2000; Bernardi, 2001; Rusko et al., 2004; Gonzalez-Rothi et al., 2015; Rybnikova et al., 2015; Serebrovska T. V. et al., 2019).

## Intermittent Hypoxic Training

Intermittent (also called interval or periodic) hypoxic training (IHT) combines episodes of hypoxia, interspersed with episodes of normoxia, hypoxia of lesser severity, hypercapnia or hyperoxia. The IHT schemes used in experiments vary greatly in the duration of the cycle, the number of hypoxic episodes and the number of days of training. From relatively short (1–10 min) episodes of hypoxia, interspersed with 1–20-min episodes of normoxia in 1 day (Cao et al., 1992) to longer daily exposures (1–12 h) for periods from 2 to 90 days (Rodriguez et al., 1999). Some authors differentiate periodic and intermittent hypoxia based on the duration of hypoxic episodes. Thus, periodic hypoxia includes sessions lasting from 20 to 30 min to several hours daily or every other day whereas intermittent hypoxia is characterized by a shorter duration (5–10 min) but a greater frequency of sessions (5–30 cycles) (Bykov et al., 2017; Saxena and Jolly, 2019). An example of periodic hypoxia is hypoxic pre- and post-conditioning techniques where rather severe episodes of hypoxia are repeated 3–6 times spaced at 24 h intervals, and such periodic hypoxia precedes (preconditioning) or follows (postconditioning) severe injurious exposure. In our well-established model, both pre- and postconditioning is performed by three trials of hypobaric hypoxia (equivalent to 5 km altitude) during 2 h each, spaced at 24 h intervals (Rybnikova et al., 2005, 2012). Such a mode of pre- and postconditioning effectively protects the brain from post-hypoxic or stress-related injury by acute mobilization of pro-adaptive gene-dependent responses (Rybnikova and Samoilov, 2015; Vetrovoy et al., 2017). IHT with short but more frequent episodes of hypoxia, on the other hand, results in progressive remodeling of major functional systems of the organism allowing adaptation to hypoxia.

An important factor in selecting the correct regime of IHT is the balance of its effectiveness and safety. Training with moderate hypoxia (9–16% O_2_) and low cycle frequency (3–15 episodes per day) most often led to a favorable effect whereas severe hypoxia (2–8% O_2_) and more episodes per day (48–2,400 per day) resulted in development of pathological conditions (Navarrete-Opazo and Mitchell, 2014). The accumulated data indicate that a “low dose” of hypoxic training can be a simple, safe, and effective method with significant therapeutic potential for clinical practice. Unlike prolonged hypoxia, which significantly reduces the initial increase in ventilation and increases the magnitude of ventilation decline, periodic hypoxia does not lead to a secondary decrease in ventilation both in experimental animals (Cao et al., 1992) and humans (Nieuwenhuijs et al., 2000). This may be due to the specific ability of periodic hypoxia to change the respiratory activity of neurons due to the induction of serotonergic-dependent long-term relief of respiratory activity (Bach and Mitchell, 1996; Turner and Mitchell, 1997), whereas prolonged hypoxia does not cause this alteration (Dwinell et al., 1997).

## Basic Technology of Normobaric Intermittent Hypoxic Training

Technically hypoxia can be achieved by breathing gas hypoxic mixtures (GHM) through special equipment (hypoxicators, rebreathers), based on the principle of return breathing. GHM with different O_2_ levels can also be generated by various technical devices including gas separation membrane installation. At the present time, the best technology is considered using devices with short-cycle oxygen adsorption from the air, eliminating the disadvantages of the membrane (Elbrus-3, Henderson tube, Epstein’s facial mask, Strelkov’s hypoxicator with chemical adsorber of CO_2_, etc.). Other equipment includes hypobaric chambers and normobaric reduced oxygen rooms (Lopata and Serebrovskaya, 2012; Serebrovskaya and Xi, 2016).

The most important aspect underlying the efficacy and safety of IHT application is its personalization (individualization). Before starting the IHT course, it is recommended to conduct a three-stage hypoxic test, during which the indicators of the functional state of the respiratory and cardiovascular systems are determined when the subject is inhaling 21% O_2_ at rest, then during inhalation of the GHM and in the near recovery period (Serebrovskaya and Xi, 2016). Several functional probes can be also applied to characterize individual tolerance to hypoxia, e.g., Shtange’s probe, amplitude of the oxyhemoglobin, etc. Dosed hypoxia, well tolerated by humans, develops in the body when breathing GHM containing at least 10% oxygen. The GHM index reflects the amount of oxygen in the mixture, for example, a GHM containing 10% oxygen is called GHM-10. Usually, the respiration with GHM is performed in a cyclic-fractional mode: breathing with a GHM—5 min, then breathing with atmospheric air—5 min (one cycle). As noted above, the number of cycles varies during one session. The total breathing time of GHM during one session is 10–15 min with a total session duration of 15–100 min (Serebrovskaya and Xi, 2016). There are variants of IHT with alternating hypoxic and hyperoxic episodes (hypoxia-hyperoxia) (Sazontova et al., 2016; Hadanny and Efrati, 2020) or hypoxic and hypercapnic episodes (hypoxia-hypercapnia) (Welch et al., 2022).

IHT has significant advantages in comparison with high-mountain therapy and barochamber hypobaric hypoxia, in particular, cost-effectiveness and accessibility of use in the clinic, the absence of negative effects of confined space (claustrophobia), and the possibility of adequate direct control of the functional state of the patient. In the training of pilots in the Royal Air Force Centre of Aviation Medicine, the breathing methods of hypoxic training have completely forced out the hypobaric hypoxia training in a barochamber since they do not have such risks as decompression sickness and barotrauma (Wrigley, 2015).

## The Mechanisms of Intermittent Hypoxic Training

Repeated episodes of hypoxia, interspaced with periods of reoxygenation, being a powerful stress factor, cause significant cumulative changes in the physiological reactions of the body. In addition to improving survival in severe hypoxia (Mayfield et al., 1994), IHT can increase the overall non-specific resistance of the body (Meerson et al., 1989; Chizhov and Strelkov, 1992). These effects are based on the phenomenon of cross-adaptation when adaptation to one stressor provides resistance to another (Meerson et al., 1996; Rybnikova et al., 2020), leading to the changes in protein expression and synthesis and in the functioning of the antioxidant systems.

The precise mechanisms of IHT have been studied rather poorly. The accumulated data allow to conclude that IHT helps to increase the efficiency of the functioning of both the respiratory system and the whole organism. It can increase production of erythropoietin (EPO) (Knaupp et al., 1992), enhance adaptive capabilities of the respiratory and cardiovascular systems in hypoxic conditions and increase the hypoxic ventilatory response *via* reduced vagal withdrawal during progressive hypoxia (Bernardi et al., 2001).

IHT results in significant improvement of the autonomic nervous system functioning, arterial stiffness, arterial endothelial function, and haemorheological function (Zembron-Lacny et al., 2020; Park et al., 2022) IHT also enhances cardiac muscle resistance to hypoxia *via* increasing the activity of the myocardial metabolic enzymes and percentage of α-myosin heavy chain (Cai et al., 2010). Activation of antioxidant enzymes and stress proteins may also be part of the mechanisms contributing to the cardioprotection of the intermittent hypoxic adaptation. Periodic hypoxia was shown to induce an increase in the concentration of heat shock proteins (HSP) in the myocardium which has a cardioprotective and antiarrhythmic effect due to intracellular changes in the ion balance (Meerson et al., 1989, 1991). Low intensity workout combined with IHT leads to a significant modulation of the immune system and inflammatory parameters, including cytokine expression, inducible nitric oxide synthase (iNOS) activity, and oxidative stress parameters (Balestra et al., 2021). IHT also resulted in increased blood superoxide dismutase (SOD) and decreased catalase (CAT) activities in an age-dependent manner (Kolesnikova et al., 2003). An increase in the efficacy of energy metabolism after hypoxic adaptation may be another mechanism for the IHT-induced cardioprotection (Gangwar et al., 2020). The possible roles of several signaling transduction pathways, including adrenoceptors, prostaglandins, and the adenosinergic system, in the beneficial effects of IHT have also been suggested (Zhuang and Zhou, 1999).

As could be expected, IHT has been shown to affect glucose metabolism (Ling et al., 2008; Cheng et al., 2011). In particular, it improves glucose tolerance and insulin response to a glucose challenge (Chen et al., 2010; De Groote et al., 2018). This effect of IHT might be related to its stimulatory action on the peptidergic neurons in the paraventricular hypothalamic nucleus (PHN) and neurons of the dorsal motor nucleus which, in turn, regulate pancreatic δ-cells and induce insulin-stimulating and insulin protective effects (Abramov, 1998).

IHT also has a direct effect on brain function and was shown to improve cerebral blood flow (Steinback and Poulin, 2016), protect cerebrovascular function (Manukhina et al., 2016), strengthen brain connectivity and increase its hypoxia tolerance (Li et al., 2016). Moreover, IHT can reduce oxidative stress caused by post-traumatic disorders at the level of carbonylated proteins and lipid peroxidation products (Manukhina et al., 2020). IHT has also been shown to suppress the cytotoxic signaling cascades activated by excess glutamate induced by ethanol withdrawal in the rat, preventing p30 activation and down-stream increase of presenilin 1 (PS1), Aβ1–40 and Aβ1–42 content in the prefrontal cortex of rats (Ryou et al., 2017). In a mouse model of Alzheimer’s disease (AD), it was shown that IHT can improve learning and memory deficits, slow Aβ accumulation in the cerebral cortex and hippocampus and increase there the levels of such neuroprotective trophic factors as erythropoietin and brain-derived neurotrophic factor (BDNF) (Ryou et al., 2021). The neuroprotective action of IHT might be associated with generation of reactive oxygen species which in turn activate an extensive defense program, including nuclear factor erythroid 2-related factor 2 (Nrf2)—a transcriptional factor regulating expression of genes encoding numerous phase II defense enzymes that collectively afford powerful antioxidant and anti-inflammatory cytoprotection (Dringen et al., 2015).

## The Therapeutic Potential of Hypoxic Training

**FIGURE 1:**
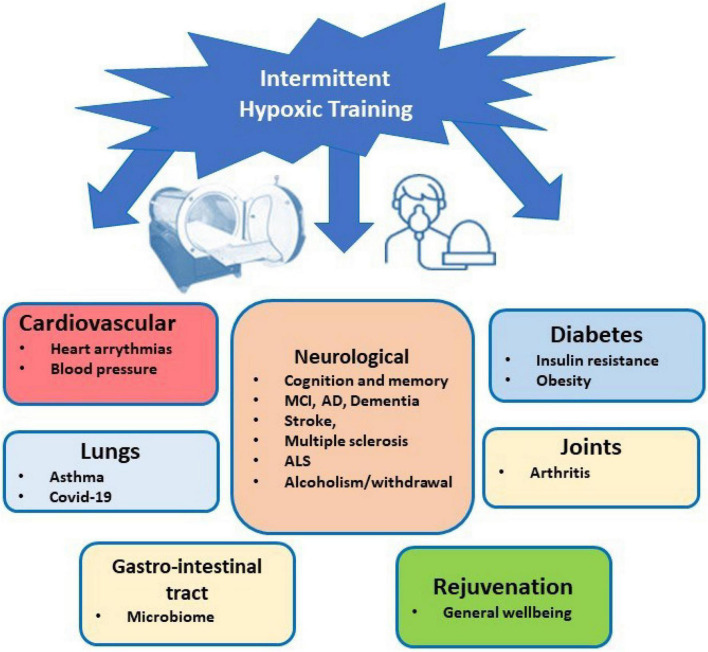
Schematic presentation of potential therapeutic benefits of IHT.

The IHT method can be recommended to treat a variety of diseases (see Figure 1). However, healthy people can use it for increasing their physical performance and stress resistance, tolerance to harmful exposures, prolongation of physical and intellectual life and prevention of dementia and neurodegeneration (Bernardi, 2001; Shatilo et al., 2008; Balestra et al., 2021). A comparative analysis of biomedical and clinical research on emerging preventative, therapeutic, and rehabilitative modality of IHT indicates that it can also have value in clinical human rejuvenation (Prokopov, 2007). Intermittent hypoxic-hyperoxic training (IHHT), when patients breathe (10–14% O_2_) for 4–7 min, followed by a 2–4-min exposure to a hyperoxic gas mixture (30–40%) through a face mask, is well tolerated by geriatric patients (up to 92 years old) and significantly improves their cognitive functions (Bayer et al., 2017). Apart from clinical application, IHT has been widely recognized in the field of military and sports medicine and is widely used for training athletes (Hamlin and Hellemans, 2007; Faiss et al., 2013; Hamlin et al., 2018).

The successful applications of IHT for the treatment of bronchial asthma, rheumatoid arthritis, anemia, neurocirculatory dystonia, and for the prevention of postoperative complications have also been described (Serebrovskaya et al., 2003). In addition to diseases directly related to hypoxia (diseases of the lungs, cardiovascular system) IHT has proven effective when used in the treatment of diseases of the gastrointestinal tract, in dermatology and hematology (Serebrovska et al., 2016). It has antidiabetic properties and in adult obese people was shown to improve weight and body mass index, fat and lean mass as well as systolic blood pressure (Camacho-Cardenosa et al., 2019).

Application of IHT can have a significant effect for prevention/treatment of the diseases caused by complications during pregnancy. Since many adult diseases have fetal origin, application of IHT in pregnancy or to the infants might prevent development of various diseases in later life (Basovich, 2013).

Accumulated evidence from ongoing preclinical research clearly demonstrates that IHT has a powerful cerebro- and neuroprotective application. IHT was also shown to be a non-invasive but powerful intervention capable of providing sustained neuroprotection during ethanol withdrawal (Jung and Mallet, 2018). It protects the brain from glutamate excitotoxicity, mitochondrial damage, oxidative stress, and amyloid β accumulation (Ryou et al., 2017). Moderate IHT *via* enhancement of cerebral oxygenation is able to improve short-term memory and attention in elderly patients with amnestic mild cognitive decline (MCI) (Wang et al., 2020). In a pilot study IHT was shown to improve cognitive functions and the levels of circulating biomarkers of AD in blood of patients with MCI suggesting that it can slow down development of AD (Serebrovska Z. O. et al., 2019). IHT was suggested to be beneficial also for treatment of patients with Parkinson’s disease (Serebrovs’ka et al., 2003) and depression (Kang et al., 2021).

With the developing COVID-19 pandemic, application of IHT for treatment of patients during the rehabilitation period has been considered as a beneficial option. The assessment of the effects of moderate-intensity IHT on health outcomes in patients recovering from COVID-19 is now under trial (Trapé et al., 2021).

## Conclusion and Perspectives for Future Research

In summary, IHT is a method elaborated for increasing human physiological defense systems, acclimatizing to high altitude, treating a variety of clinical conditions and training of sport athletes. Based on the current data, it can be assumed that training with periodic hypoxia might be a powerful, non-invasive tool to achieve reliable and stable neuroprotection. IHT, similarly to hypoxic pre- and postconditioning, can cause proadaptive modifications of the glucocorticoid system and stimulate production of the neurotrophins, in particular BDNF (Rybnikova et al., 2015) but to date no detailed studies have addressed these important aspects. The disclosure of IHT molecular mechanisms will contribute to the successful realization of the therapeutic and health-promoting potential of this method for the benefits of human wellbeing and mental health.

## Author Contributions

All authors listed have made a substantial, direct, and intellectual contribution to the work, and approved it for publication.

## Conflict of Interest

The authors declare that the research was conducted in the absence of any commercial or financial relationships that could be construed as a potential conflict of interest.

## Publisher’s Note

All claims expressed in this article are solely those of the authors and do not necessarily represent those of their affiliated organizations, or those of the publisher, the editors and the reviewers. Any product that may be evaluated in this article, or claim that may be made by its manufacturer, is not guaranteed or endorsed by the publisher.
